# Useful Flies

**DOI:** 10.3390/ijms20040871

**Published:** 2019-02-18

**Authors:** Beat Suter

**Affiliations:** Institute of Cell Biology, University of Bern, Baltzerstrasse 4, 3012 Bern, Switzerland; beat.suter@izb.unibe.ch; Tel.: +41-31-631-4715

Many molecular and cellular mechanisms that drive the physiological functions of cells or control the development of an animal are well conserved between vertebrates and insects. This conservation makes the genetically and experimentally tractable *Drosophila* a valuable model to study human development and diseases. The high similarity of these processes is illustrated by the fact that close to two out of three human disease genes have *Drosophila* counterparts that are thought to be functional homologs.

The availability of extensive resources and sophisticated genetic tools allows researchers to efficiently generate *Drosophila* disease models for numerous disorders with the goal of identifying the etiology of these human diseases. Knowledge about a disease pathway often points to novel possible therapeutic targets, and whole organism screening for compounds that interact with the pathway. Because *Drosophila* allows researchers to adjust the genetic background, whole organism *Drosophila* disease models will also play an important role in the future of personalized medicine.

Major granting agencies all over the world have recognized the tremendous value of model organisms, including *Drosophila*, for the study of human diseases. Accordingly, they have started programs to foster the interaction between researchers studying human patients and researchers studying neurobiological, developmental and physiological processes in vertebrate and non-vertebrate model systems. Oriel and Lasko [1] provide an overview of these programs, highlighting the role that *Drosophila* plays in understanding human diseases and how the fly is used for exploiting potential therapeutic targets. In addition to granting agencies, human disease research using *Drosophila* is also facilitated by databases and websites that link the relevant information and display them for interested researchers. Flybase (http://flybase.org) compiles the human disease model data for *Drosophila*, and efforts from around the world continuously produce additional supporting information. In this issue, Wei et al. [2] present their database that links human miRNAs and their disease-related target genes with predicted *Drosophila* counterparts. 

Several reviews cover the present knowledge on *Drosophila* research designed to reveal pathogenic mechanisms and to explore possible therapeutic strategies. Souidi et al. [3] have reviewed the field of Myotonic Dystrophy Type 1. Dietary effects and consequences of obesity have been studied extensively in flies and different aspects of these studies are covered in this issue as well. Lian et al. [4] reviewed such effects on gut metabolic homeostasis, immune function and aging. Gáliková and Klepsatel [5] discuss the effects of obesity on life span, physiology and aging, and Warr et al. [6] explore links between dietary conditions, obesity, diabetes and cancer. *Drosophila* is being used heavily for cancer related research and Trivedi and Starz-Gaiano [7] have focused their review on the Jak/Stat signaling pathway and its role in cell differentiation, migration, proliferation and cancer, and on the value of screening for drugs using *Drosophila.* The article by Powers and Srivastava [8] discusses the value of two fly models for cell migration and metastasis. One model was introduced some time ago and the other is a novel one.

Because of its relative simplicity and the elaborated genetic tools available, the fly nervous system, including the brain, has been another main focus of *Drosophila* research relating to human disease. Lye and Chtarbanova [9] present a review on innate immunity and inflammatory reactions with a focus on its value and dangers for the brain. Kasture et al. [10] reviewed the knowledge about dopaminergic and serotonergic neurotransmission in the fly brain. They point out how this knowledge helps in understanding human health-related neuronal functions and disease mechanisms, and also in finding potential ways to treat such diseases. Many other contributors have focused their reviews on various aspects of neurodegeneration. Nagoshi [11] reviewed the contribution of *Drosophila* to identifying risk factors and providing mechanistic insights into the pathogenesis of sporadic Parkinson’s disease. She also discusses the potential of *Drosophila* for the development of preclinical animal models. Rosas-Arellano et al. [12] reviewed the work on Huntington’s disease and what improves this disease phenotype, and Monnier et al. [13] discuss how *Drosophila* research helped understanding of the development and progression of Friedreich’s ataxia and how these results led to the development of ideas of what drugs could ameliorate the condition. Vandal et al. [14] present the knowledge gained about Frontotemporal dementia with a special focus on a truncation mutation in the *CHMP2B* locus that affects the endosomal–lysosomal pathway and causes neurodegeneration.

This special issue also contains two research papers in which *Drosophila* researchers address human health issues in the fruit fly. Using the *Drosophila* eye, Poon et al. [15] investigated how *Src* and oncogenic *ras* cooperate in epithelial tumor formation. The second research article relates to neurodegeneration. In different neurodegenerative diseases in humans, the nuclear RNA binding protein TDP-43 (TBPH in flies) is found in unusual cytoplasmic hnRNP structures that have been linked to ALS and FTD. Lo Piccolo et al. [16] now show that very similar structures also appear in the cytoplasm of *Drosophila* tissue if the *ISWI* chromatin modifier is mutated. They used the *Drosophila* system to study the formation of these cytoplasmic inclusions to obtain insights into the etiology of neurodegeneration associated with cytoplasmic TDP-43/TBPH inclusions. 

## References

[1] Oriel C., Lasko P. (2018). Recent Developments in Using *Drosophila* as a Model for Human Genetic Disease. Int. J. Mol. Sci..

[2] Wei G., Sun L., Qin S., Li R., Chen L., Jin P., Ma F. (2018). Dme-Hsa Disease Database (DHDD): Conserved Human Disease-Related miRNA and Their Targeting Genes in *Drosophila melanogaster*. Int. J. Mol. Sci..

[3] Souidi A., Zmojdzian M., Jagla K. (2018). Dissecting Pathogenetic Mechanisms and Therapeutic Strategies in *Drosophila* Models of Myotonic Dystrophy Type 1. Int. J. Mol. Sci..

[4] Lian T., Wu Q., Hodge B.A., Wilson K.A., Yu G., Yang M. (2018). *Drosophila* Gut—A Nexus Between Dietary Restriction and Lifespan. Int. J. Mol. Sci..

[5] Gáliková M., Klepsatel P. (2018). Obesity and Aging in the *Drosophila* Model. Int. J. Mol. Sci..

[6] Warr C.G., Shaw K.H., Azim A., Piper M.D.W., Parsons L.M. (2018). Using Mouse and *Drosophila* Models to Investigate the Mechanistic Links between Diet, Obesity, Type II Diabetes, and Cancer. Int. J. Mol. Sci..

[7] Trivedi S., Starz-Gaiano M. (2018). *Drosophila* Jak/STAT Signaling: Regulation and Relevance in Human Cancer and Metastasis. Int. J. Mol. Sci..

[8] Powers N., Srivastava A. (2018). The Air Sac Primordium of *Drosophila*: A Model for Invasive Development. Int. J. Mol. Sci..

[9] Lye S.H., Chtarbanova S. (2018). *Drosophila* as a Model to Study Brain Innate Immunity in Health and Disease. Int. J. Mol. Sci..

[10] Kasture A.S., Hummel T., Sucic S., Freissmuth M. (2018). Big Lessons from Tiny Flies: *Drosophila melanogaster* as a Model to Explore Dysfunction of Dopaminergic and Serotonergic Neurotransmitter Systems. Int. J. Mol. Sci..

[11] Nagoshi E. (2018). *Drosophila* Models of Sporadic Parkinson’s Disease. Int. J. Mol. Sci..

[12] Rosas-Arellano A., Estrada-Mondragón A., Piña R., Mantellero C.A., Castro M.A. (2018). The Tiny *Drosophila Melanogaster* for the Biggest Answers in Huntington’s Disease. Int. J. Mol. Sci..

[13] Monnier V., Llorens J.V., Navarro J.A. (2018). Impact of *Drosophila* Models in the Study and Treatment of Friedreich’s Ataxia. Int. J. Mol. Sci..

[14] Vandal S.E., Zheng X., Ahmad S.T. (2018). Molecular Genetics of Frontotemporal Dementia Elucidated by *Drosophila* Models—Defects in Endosomal–Lysosomal Pathway. Int. J. Mol. Sci..

[15] Poon C.L., Brumby A.M., Richardson H.E. (2018). *Src* Cooperates with Oncogenic *Ras* in Tumourigenesis via the JNK and PI3K Pathways in *Drosophila* epithelial Tissue. Int. J. Mol. Sci..

[16] Lo Piccolo L., Bonaccorso R., Attardi A., Li Greci L., Romano G., Sollazzo M., Giurato G., Ingrassia A.M.R., Feiguin F., Corona D.F.V. (2018). Loss of ISWI Function in *Drosophila* Nuclear Bodies Drives Cytoplasmic Redistribution of *Drosophila* TDP-43. Int. J. Mol. Sci..

